# Cerebral haemodynamics and intracranial pressure during haemorrhagic shock and resuscitation with total endovascular balloon occlusion of the aorta in an animal model

**DOI:** 10.1007/s00068-024-02646-0

**Published:** 2024-10-25

**Authors:** Sam Er Bader, C. Brorsson, N. Löfgren, F. Löfgren, P-J. Blind, N. Sundström, M. Öman, M. Olivecrona

**Affiliations:** 1 Department of Surgery, Faculty of Medicine and Health, Örebro University, Örebro, Sweden; 2 Department of Surgical and Perioperative Sciences, Anaesthesia and Intensive Care, Umeå University, Umeå, Sweden; 3 Department of Surgical and Perioperative Sciences; Surgery, Umeå University, Umeå, Sweden; 4 Department of Radiation Sciences, Radiation Physics, Biomedical Engineering, Umeå University, Umeå, Sweden; 5 Department of Neurosurgery, Faculty of Medicine and Health, Örebro University, Örebro, Sweden

**Keywords:** Resuscitative endovascular balloon occlusion of the aorta, REBOA, Haemorrhagic shock, ICP, Cerebral autoregulation

## Abstract

**Purpose:**

To assess changes of cerebral haemodynamic and intracranial pressure (ICP) in animals, with or without elevated ICP, during controlled haemorrhagic shock and resuscitation with Total REBOA (tREBOA).

**Method:**

In 22 anaesthetized and normoventilated pigs, after placement of catheters for monitoring invasive proximal blood pressure (pMAP), ICP, and vital parameters, and 60 min stabilisation phase, a controlled haemorrhagic shock (HS), was conducted. In 11 pigs (EICPG), an elevated ICP of 25–30 mmHg at the end HS was achieved by simulating an epidural mass. In 11 pigs (NICPG), the ICP was normal. tREBOA was then applied for 120 min. The changes of pMAP and ICP were followed, and cerebral perfusion pressure (CPP) calculated. The integrity of the autoregulation was estimated using a calculated Modified-Long Pressure Reactivity Index (mL-PRx).

**Results:**

After stabilisation, hemodynamics and physiological parameters were similar and normal in both groups. At the end of the HS, ICP was 16 mmHg in NICPG vs. 32 in EICPG (*p* = 0.0010). CPP was 30 mmHg in NICPG vs. 6 mmHg in EICPG (*p* = 0.0254). After aorta occlusion CPP increased immediately in both groups reaching after 15 min up to104 mmHg in NICPG vs. 126 mmHg in EICPG. Cerebrovascular reactivity seems to be altered during bleeding and occlusion phases in both groups with positive mL-PRx. The alteration was more pronounced in EICPG, but reversible in both groups.

**Conclusion:**

tREBOA is lifesaving by restoration the cerebral circulation defined as CPP in animals with HS with normal or elevated ICP. Despite the observation of short episodes of cerebral autoregulation impairment during the occlusion, mainly in EICPG, tREBOA seems to be an effective tool for improving cerebral perfusion in HS that extends the crucial early window sometimes known as the “golden hour” for resuscitation even after a traumatic brain injury.

## Introduction

Aortic occlusion (AO) is a well-known procedure. Long used in cardiovascular surgery, it has also been practised in massive non-compressible haemorrhage to help achieve distal control and preserve adequate cardiac output to the upper body and brain. In uncontrolled haemorrhage, AO has traditionally been performed by either manual, external compression of the abdominal aorta, as in postpartum haemorrhage, or invasively by means of thoracotomy with aortic cross-clamping. Less invasive is the technique of endovascular balloon occlusion of the aorta, first used during the Korean War in 1954 [1].

In uncontrolled bleeding with haemorrhagic shock (HS), a balloon catheter is inserted into the aorta through the femoral artery. The balloon is then inflated to occlude the aorta, thereby maximising perfusion to the organs proximal to the balloon (the brain, heart, and lungs) and minimising distal flow. This mitigates the haemorrhage until definite haemostasis can be achieved [2–6].

Non-compressible haemorrhage resulting in HS is frequently associated with traumatic brain injury (TBI) in trauma patients, and may need aggressive interventions, such as aortic cross-clamping or resuscitative endovascular balloon occlusion of the aorta (REBOA), to restore haemodynamic stability and save the brain from further hypoperfusion. TBI has been regarded as a possible contraindication to AO, as theoretically the supraphysiological proximal mean arterial pressure (pMAP) observed after AO, may exacerbate intracranial haemorrhage and cerebral oedema [7–14].

Experimental animal studies have reported an initial, sudden, and short-lived elevation of intracranial pressure (ICP) after AO. During occlusion, a rise in pMAP, monitored in the carotid artery, to supraphysiological levels has been reported. In most of these studies, the thoracic aorta was totally occluded by cross-clamping in euvolemic animals without brain injury. The studies also show an increase in brain-tissue oxygen (p_br_O_2_), carotid blood flow and CBF [15–21].

In the normal brain, cerebral autoregulation (CA) serves to maintain a continuous and stable cerebral blood flow (CBF) and cerebral blood volume (CBV), regardless of fluctuations in systemic blood pressure. This autoregulation maintains a stable CBF when mean arterial pressure (MAP) is in the range of 50–150 mmHg. Beyond these limits, CBF varies passively with changes in MAP [22, 23]. The physiological haemodynamic response in severe hypotension in the intact brain is a tone alteration in vessels resistance causing a transient vasodilatation and hyperperfusion. In injured brain with oedema and/or ICP elevation, where neurovascular function is impaired and CA capacity is reduced, a severe hypotension induces initial vasoconstriction and severe hypoperfusion and ischemia [24]. One of the most used tools for evaluation of cerebrovascular reactivity and CA in injured brain is the pressure reactivity index (PRx). It’s a moving Pearson correlation coefficient between MAP and ICP waves [25–27]. PRx reflects the cerebral vessels resistance-capacity to adjust their diameter when CPP changes. A positive PRx value indicates CA disturbance. However, a value over 0.2 is associated with increase in mortality in TBI and intracerebral bleeding [28–32].

Understanding the haemodynamic effects and consequences of AO in a brain with or without elevated ICP is essential for improving trauma management [8, 12, 33–36].

A few recent studies have explored the effect of REBOA on cerebral haemodynamic (CH) with and without brain injury [37–42]. They were unable to establish that using REBOA in animals in HS and with TBI worsened the brain injury, although ICP and pMAP rose significantly [20, 38, 42].

Edwards et al. recently compared the effect of total REBOA (tREBOA) and partial REBOA (pREBOA) in animals with HS and elevated ICP (> 20 mm Hg). While the REBOA was activated, cerebral perfusion pressure (CPP) was elevated in both groups, though higher in the tREBOA group. However, after the deflation of the REBOA in what is known as the “resuscitation phase”, the opposite findings were noted [43].

The purpose of this study is to assess CH, ICP and CA during controlled HS and resuscitation, using tREBOA in an animal model.

## Materials and methods

### Ethics

The study was approved by the Animal Experimental Ethics Committee at Umeå University, Sweden (A 32–19) and conducted in accordance with Directive 2010/63/EU on protection of animals used for scientific purposes, and the *Guide for the Care and Use of Laboratory Animals*, National Research Council, Washington, DC, USA, 1996.

### Study overview

We studied two groups of animals. The first group (pig 1–11) had normal ICP (Normal ICP Group, NICPG), while the second group (pig 12–22) had elevated ICP (Elevated ICP Group, EICPG).

**Fig. 1:**
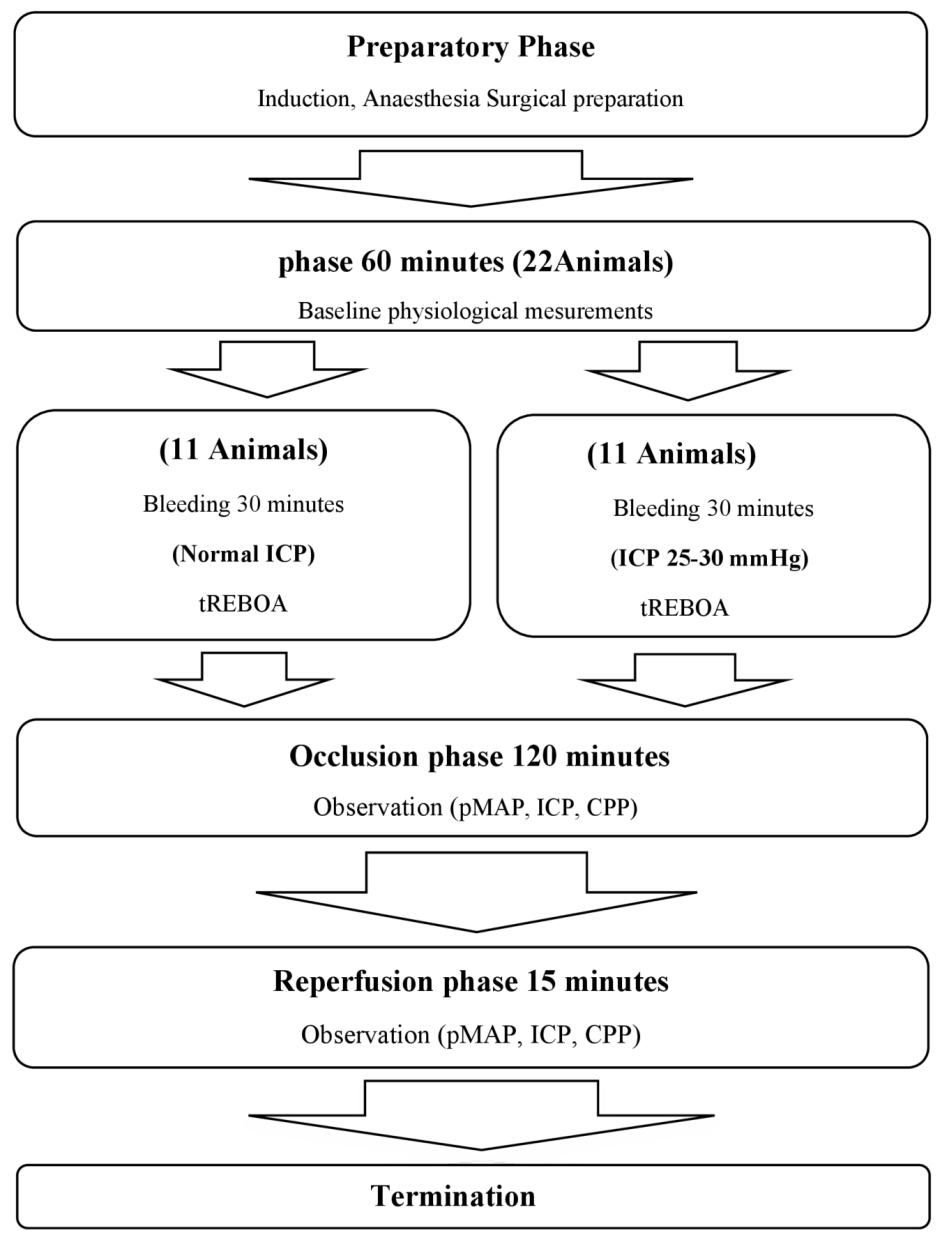
Experimental design and timeline in minutes

The study comprised six phases: preparation, stabilisation, bleeding (with or without ICP elevation), aortic occlusion, reperfusion, and termination (Fig. 1).

### Animal preparation

#### Anaesthesia

Anaesthesia was performed as previously described by Abrahamsson and colleagues [44]. Briefly, animals were premedicated with intramuscular administration of ketamine 10 mg/kg (Ketalar^®^; Pfizer, Morris Plains, NJ, USA), xylazine 20 mg/kg (Rompun vet Bayer AB) and atropine sulphate 0·05/mg kg (Atropine, NM; Pharma, Stockholm, Sweden).

After cannulation of the right-ear vein, anaesthesia was induced by an intravenous bolus dose of 10 mg/kg sodium pentobarbital (Pentobarbital natrium; Apoteksbolaget, Stockholm, Sweden). Fentanyl at 20 µg/kg per hour (Fentanyl; Braun, Melsungen, Germany), midazolam at 0.3 mg/kg per hour (Dormicum; Roche, Basel, Switzerland) and sodium pentobarbital at 5 mg/kg per hour was used for maintenance of anaesthesia.

The animals were tracheotomized (7.0 OD endotracheal tube; Rusch, Kernen, Germany) and mechanically normoventilated (Evita 4, Dräger, Laubach, Germany) with an air mixture containing 30% oxygen, and guided by repeated arterial blood gas analyses (ABL 5 autoanalyzer; Radiometer, Copenhagen, Denmark).

In the first hour, 1000 ml of Ringer’s acetate (Pharmacia, Upjohn, Sweden) was infused, followed by continuous infusion starting at 15 ml/kg per hour. The infusion rate was adjusted during the experiment to maintain a central venous pressure (CVP) of 5–10 mmHg.

### General monitoring and surgical preparation

Heart rate (HR) was measured by means of an electrocardiogram and saturation through pulse oximetry (GE HealthCare, Chicago, Illinois, USA) on the right ear. Using the cut-down technique, a central venous catheter was inserted in the left internal jugular vein and an arterial line in both external left carotid arteries and the left femoral artery. CVP and arterial blood pressure (ABP) were measured continuously (Abbott, Chicago, Illinois, USA) at the level of the left atrium.

A suprapubic urinary catheter was placed in the bladder. The REBOA catheter (Vingmed AB, Järfälla, Sweden) was inserted in the right femoral artery using the cut-down technique. Confirmation of adequate placement of the balloon above the diaphragmatic crus, in the thoracic descending aorta (zone 1), was obtained under tactile guidance. ABP and saturation were documented every five minutes.

### Cerebral monitoring and ICP elevation

After anaesthesia and overall monitoring were established, the animal was placed in a prone position. The head was shaved, cleaned, and disinfected. The cranial bone was exposed with a paramedian incision 6 cm long on the left side. A 3 mm burr hole was drilled frontally. Next, the dura was opened sharply, and the haemostasis secured by diathermy. A four-lumen bolt (H QFlow 500 Titanium, Hemedex, Waltham, MA, USA) was placed in the burr hole.

An intraparenchymal catheter measuring ICP and temperature (PSO-PTT, Sophysa, Orsay, France) was calibrated according to the manufacturer’s instructions, introduced through the bolt to a depth of 10 mm into the brain and connected to an ICP monitor (PSO-4000 Pressio 2, Sophysa, Orsay, France). ICP was continuously measured and manually recorded every five minutes. CPP was calculated according to the formula CPP = MAP − ICP.

To obtain an ICP elevation in EICPG and simulate an acute epidural haematoma, an extra 6 mm burr hole was drilled in the right side of the skull, where a Foley catheter (Ch12) was inserted between the intact dura and the bone.

### Study design and experimental protocol (Fig. 1)

#### Stabilisation phase (60 min)

The animals were placed in a supine position at a table inclination of 15°. Consequently, the zero level of the ABP equals that of the ICP, making correction of the CPP redundant.

Cefuroxime 750 mg (MIP Pharma GmbH, Blieskastel, Germany) was administered intravenously, and the stabilisation phase of 60 min commenced. The end of the stabilisation phase (T–30 min) was adopted as the reference point for all measurements during the experiment.

### Bleeding phase (with or without ICP elevation; 30 min)

An intravenous dose of heparin 5000 IE (Heparin LEO Pharma AB; Malmö, Sweden) was given before the bleeding phase. During 30 min, 40% of the animal’s estimated blood volume was drained continuously from the left femoral artery catheter with a syringe. The blood volume (L) of the animal was estimated at 8% of body weight. The blood loss speed in ml/minute = Estimated blood volume / 30.

During this phase, Adrenalin (Mylan, Canonsbury, Pennsylvania, USA) was used as needed to mimic the physiological stress response and keep the MAP at > 40 mmHg.

In the EICPG, an ICP elevation prior to AO was achieved during the bleeding phase by successively filling the epidurally placed Foley catheter with saline to mimic a growing epidural haematoma. We aimed at an ICP of 25–30 mmHg at the end of the bleeding phase.

### Occlusion phase (120 min)

Once hypovolemia was achieved, the REBOA catheter was inflated with saline until pressure waves in the femoral artery disappeared (T = 0 min) and kept fully inflated for two hours.

After REBOA was inflated, 500 ml 6% hydroxyethyl starch in sodium chloride (Voluven^®^ Fresenius Kabi, Homburg, Germany) was administered intravenously to mimic prehospital treatment of hypovolemia.

### Reperfusion phase (15 min) and termination

After completion of the REBOA phase, the aortic occlusion balloon was deflated at a rate of 1 ml/min until proximal and distal blood pressures were equalised. The reperfusion phase lasted 15 min whereafter the animal was euthanized with a lethal intravenous injection of sodium pentobarbital 400 mg and potassium chloride 40 mmol (Kaliumklorid; B. Braun medical AB, Danderyd, Sweden).

### Evaluation of cerebral autoregulation

To estimate brain vessel response and changes in autoregulation, we calculated a pressure reactivity index (PRx) using the correlation between pMAP and ICP, in analogy with Long PRx, as proposed by Sánchez-Porras et al. in 2012 [31]. They used the correlation between 20 pMAP and ICP measurements at one-minute intervals. Analogously, we calculated the correlation of six consecutive pMAP and ICP values at five-minute intervals, modified Long-PRx (mL-PRx), due to the time resolution of the recorded data, and chose to interpret mL-PRx ≤ 0 as a sign of preserved cerebral reactivity.

### Statistics

The data was tested using Shapiro-Wilk test. A large amount of data was found not to be normally distributed. Thus, non-parametric tests were used, and the data was presented by median values and interquartile ranges (IQR). The Wilcoxon Rank Sum Test was used to determine whether there were any statistically significant differences between the NICPG and EICPG, and the Wilcoxon Signed Rank Test was applied to investigate possible differences within the groups. P values are reported to four decimal places. Statistical analysis was performed in JMP 16.2.0 (SAS Institute Inc. Cary, NC, USA).

## Results

Twenty-two pigs (16 female and 6 male) with a median weight of 47.5 kg (IQR 44–52) were included in the study. All 22 were kept alive until the end of the experiment and included in the analysis.

Haemodynamic and basic physiology at baseline (T − 30 min) were similar and within the normal range in both groups.

Median (IQR) pMAP at the start of bleeding phase (T -30 min) was 94 (78–102) mmHg in NICPG, against 99 (75–107) mmHg in EICPG (*p* = 0.0532). Median ICP (IQR) in NICPG was 17 (14–23) mmHg against 20 (15–21) mmHg in EICPG (*p* = 0.9737). However, median (IQR) CPP was higher in EICPG, 80 (60–85) mmHg vs. 71 (66–84) mmHg in NICPG (*p* = 0.0511).

### Proximal mean arterial pressure (pMAP)

**Fig. 2:**
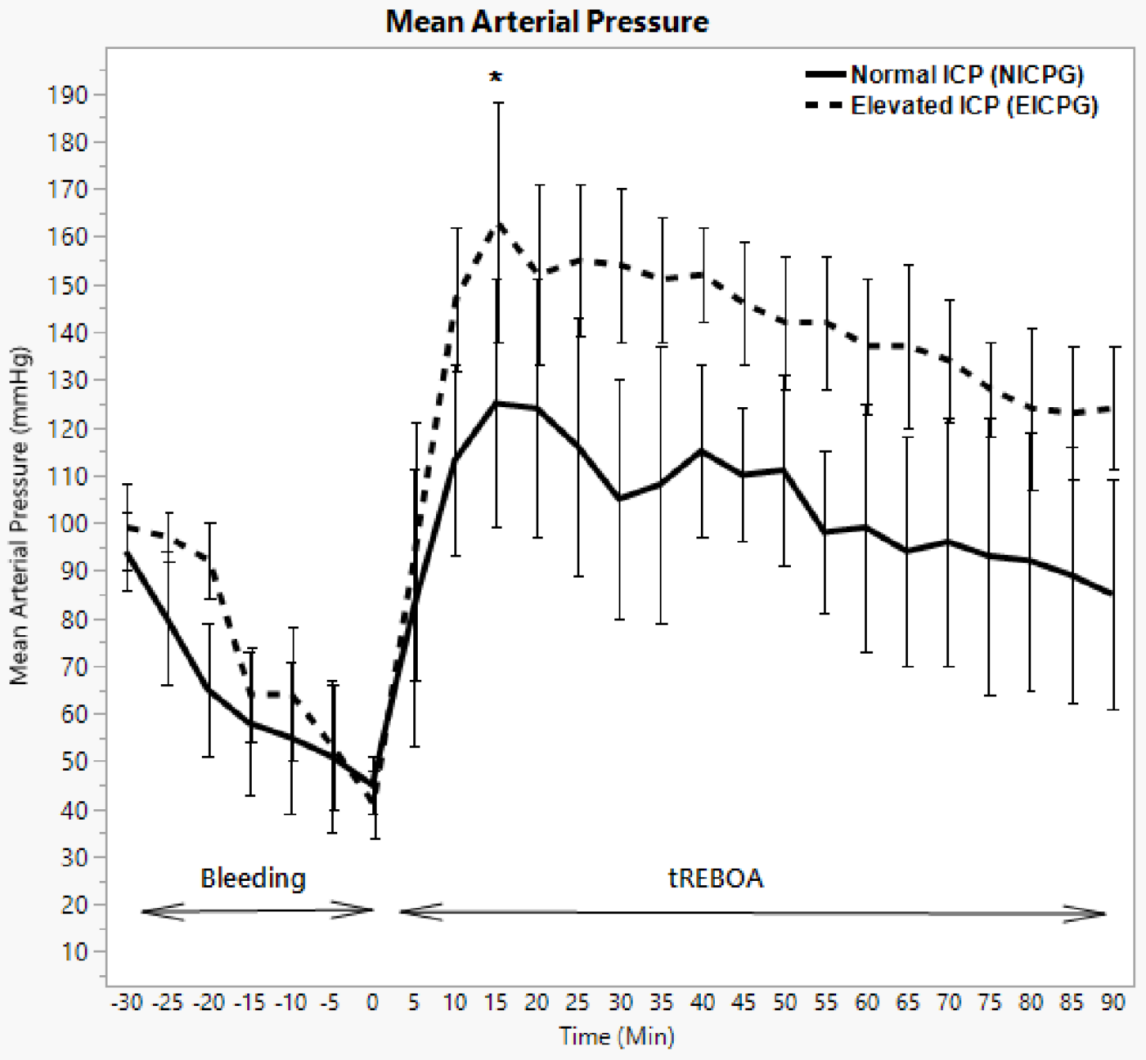
Mean Arterial Pressure in NICPG and EICPG. Error bars show median absolute deviation. * = significant difference between the two groups with P- value < 0.05

During bleeding phase, pMAP decreased gradually in NICPG from median (IQR) 94 (78–102) mmHg to 45 (38–49) mmHg (*p* = 0.0010) prior to the occlusion. After occlusion, pMAP reached a maximum of 125 (99–162) mmHg (*p* = 0.0010), at T15, and then gradually decreased to 85 (61–109) mmHg (*p* = 0.0010) at T 90 (Fig. 2).

In EICPG, pMAP decreased during the bleeding phase from 99 (75–107) mmHg to 41 (34–50) mmHg (*p* = 0.0020). After occlusion, pMAP reached a maximum of 163 (137–188) mmHg (*p* = 0.0010) at T 15 min. The maximum increase in pMAP compared with baseline MAP (T-30) was 64 mmHg (*p* = 0.0020). At T 90 min, pMAP reached 124 (103–137) mmHg, compared with 41 (IQR 34–50) mmHg (*p* = 0.0010) at T 0 min (Fig. 2).

A statistically significant difference in pMAP between the two groups was observed during the occlusion, mainly between T 25 and T 90 min.

### Intracranial pressure (ICP)

**Fig. 3:**
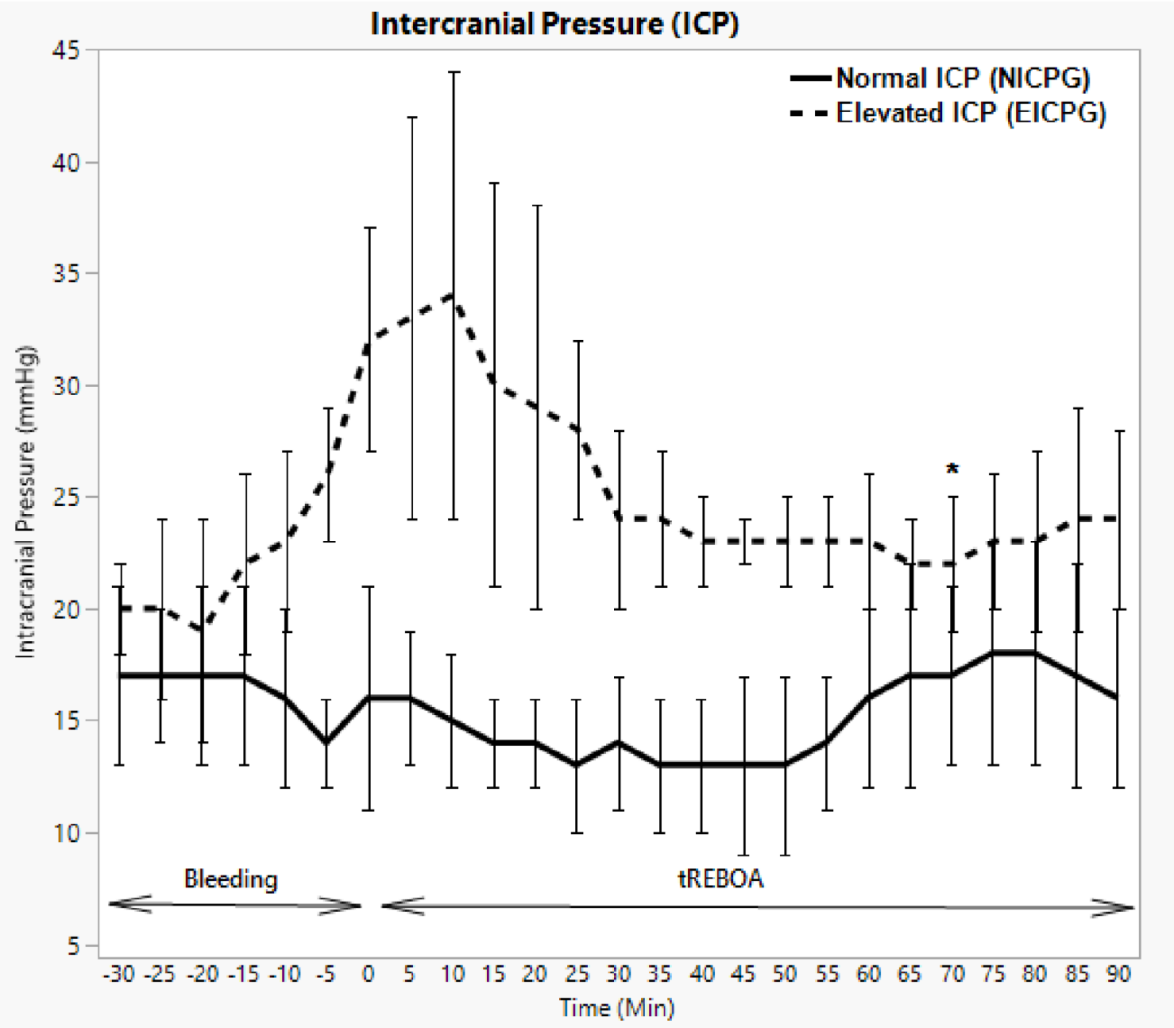
Intracranial pressure in NICPG and EICPG. Error bars show median absolute deviation. * = significant difference between the two groups with P value < 0.05

ICP was generally stable in NICPG. The median ICP in EICPG was 32 mmHg (IQR 25–34) at the end of the bleeding phase. The ICP elevation continued after the activation of the tREBOA in EICPG, reaching its maximum 10 min after the AO, with a median ICP of 34 mmHg (IQR 26–65; *p* = 0.2686), after which it decreased gradually without returning to baseline levels (Fig. 3).

### Cerebral perfusion pressure (CPP)

**Fig. 4:**
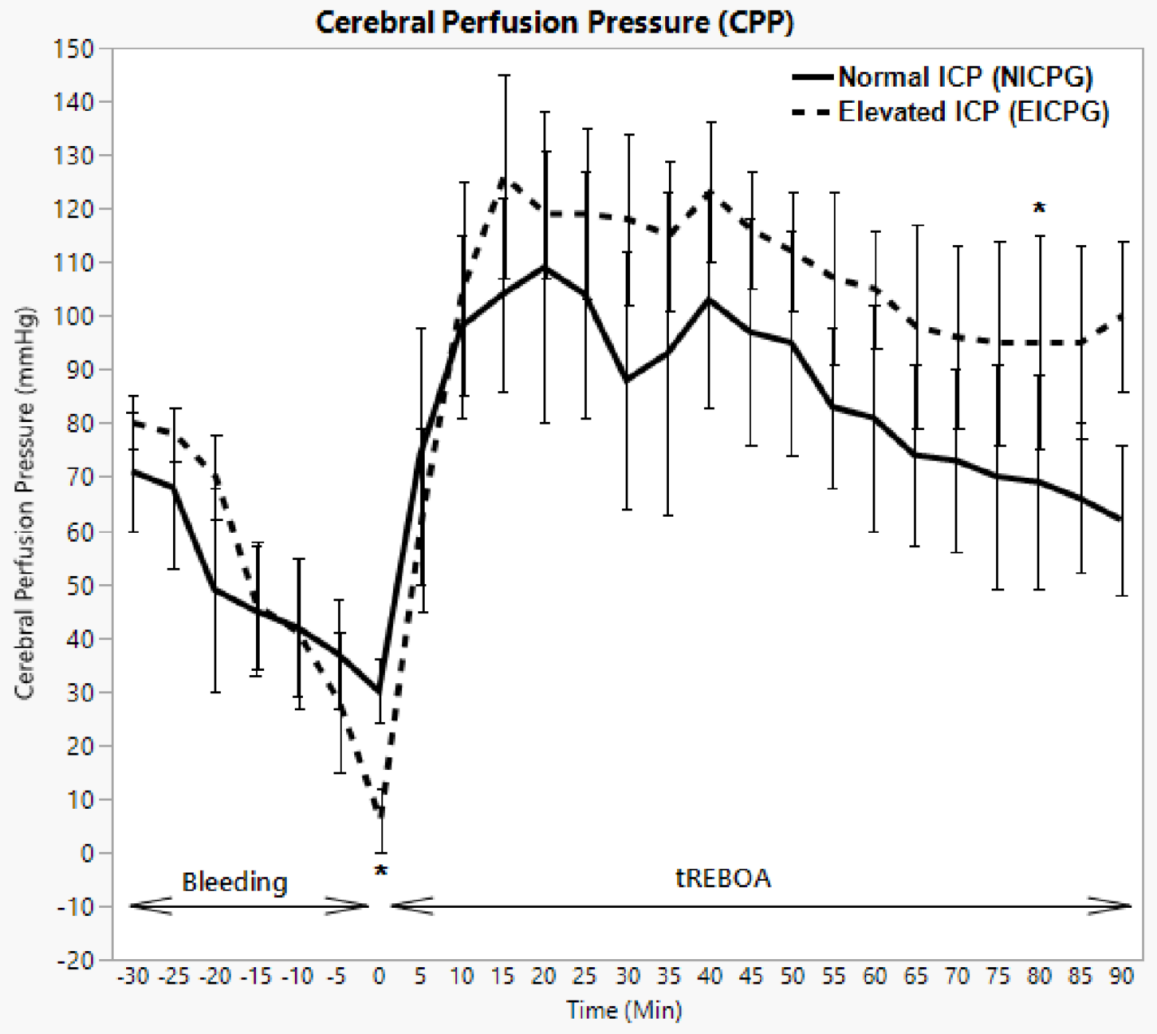
Cerebral perfusion pressure in NICPG and EICPG. Error bars show median absolute deviation. * = significant difference between the two groups with P value < 0.05

In NICPG the haemorrhage caused a decrease in CPP from median (IQR) 71 (66–84) mmHg at the baseline to 30 (22–36) mmHg (*p* = 0.0010) by the end of the bleeding phase. After the occlusion, CPP increased to a high level and reached a maximum of 104 (90–151) mmHg at T 15 min (*p* = 0.0010) before it started to return to the baseline at T 90 min (Fig. 4).

In EICPG, in contrast, CPP reached the extremely low level of six (IQR 2–21) mmHg by the end of the bleeding phase, against 80 (60–85, *p* = 0.0020).

After activation of the tREBOA the median (IQR) CPP increased to 126 (99–133) mmHg at T15 (*p* = 0.0010) (Fig. 4).

At the end of the bleeding phase and prior to balloon inflation, a difference in CPP between the two groups (*p* = 0.0254) was observed. However, no difference was observed thereafter until T 80.

### Autoregulation

**Fig. 5:**
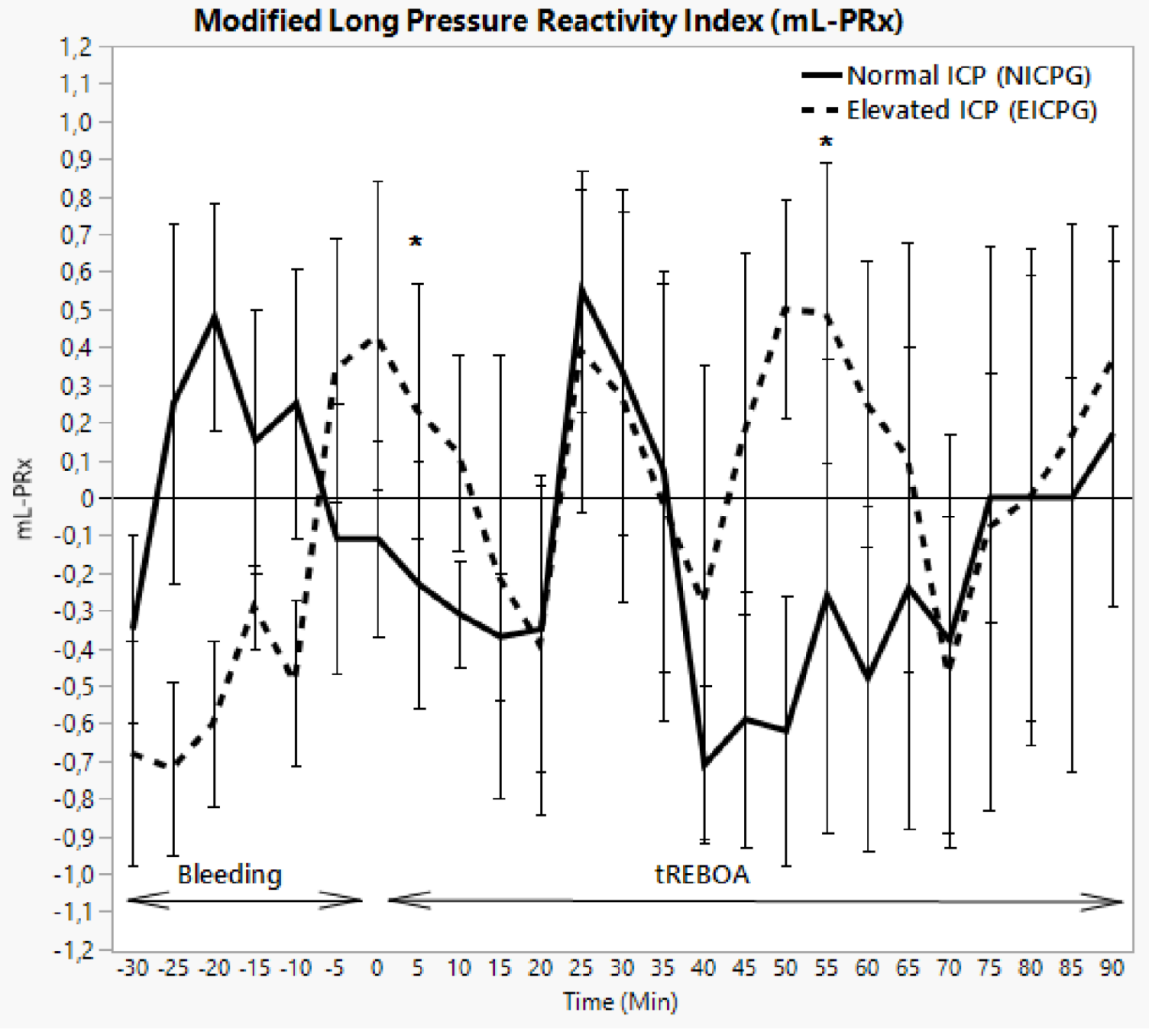
Intracranial pressure in NICPG and EICPG. Error bars show median absolute deviation. * = significant difference between the two groups with P value < 0.05

The autoregulation estimates are shown in Fig. 5. In NICPG, an elevation of mLPRx and a slight deterioration in CA were seen during the initial bleeding, appeared to be resolved over time even before AO. A second phase of autoregulatory impairment was evident approximately between 20 and 40 min of the REBOA activation, after which autoregulation seemed to be restored.

In EICPG, the same pattern was observed but the CA continued to deteriorate until the inflation of REBOA. After AO and normalisation of CA for ten minutes, we observed two more reversible episodes of autoregulatory compromise during the occlusion time (Fig. 5).

## Discussion

### Proximal mean arterial pressure (pMAP)

In our study, we confirmed the earlier findings that tREBOA is an effective tool for raising pMAP in HS from low to normal or even supranormal levels in both animals and humans [38, 45–47].

Applying tREBOA in animals with HS increases pMAP to adequate levels in NICPG. This would serve to maintain CBF, thus avoiding cerebral hypoperfusion. We also observed that tREBOA can induce a supraphysiological pMAP in EICPG, which may be harmful to the brain [45, 46, 48–51].

In EICPG compared with NICPG, we observed an immediate increase in pMAP after AO, reaching the maximum after 15 min. In NICPG the same pattern was observed with an initial increase in pMAP and after peaking, a decrease over time. The maximum increase in pMAP in EICPG can theoretically be so high as to compromise CA, i.e., it may exceed 150 mmHg. This difference in pMAP between the two groups can be explained at least partly by a physiological compensatory response of pMAP to ICP elevation, aimed at maintaining an adequate CBF [21].

#### Intracranial pressure (ICP)

Patients with severe trauma and haemorrhagic shock do not seldom present with intracranial injuries which may lead to life-threatening intracranial hypertension [52].

Our model tries to simulate such a situation, mimicking patients suffering from a closed head injury resulting in an acute rise in ICP as in the case of epidural or subdural haematoma.

In NICPG, a moderate decreasing in ICP observed by the end of bleeding phase. However, ICP was stable and within the normal range during AO.

In numerous studies, elevated ICP during aorta cross-clamping has been observed, mainly in the context of normovolemia but also in animals in HS [16, 20, 21, 38, 42, 53, 54]. The elevation in MAP after AO induces cerebral vasoconstriction in the intact brain, with a decrease in ICP and CBV, indicating preservation of CA [55].

In the EICPG, a further elevation of the ICP occurred after AO, and lasted longer before the ICP returned to the baseline values. This delay might be due to a temporarily altered or compromised CA. In the injured brain where cerebrovascular reactivity is impaired, MAP elevation will cause an increase in ICP due to a passive response of the cerebral resistance vessels [55].

We propose an interpretation of the return of ICP to the baseline ICP in both groups as a sign of slowly recovering CA. Edward et al. show in there 2022 study, as we do, an increase in CPP during the use of REBOA which theoretically could be harmful to the brain. Further, they show that during resuscitation i.e., deflation of the balloon, and statistically significant rise in lactate which could be a cause of further brain damage. Unfortunately, in our study, we haven’t studied the change in lactate [43].

The mechanism underlying ICP elevation during AO remains unclear. However, the progression of reversible ICP elevation during AO is too rapid to be related to changes in the volume of cerebrospinal fluid due to increased production or decreased reabsorption. The more plausible explanation of the transient rise in ICP is a rise in CBV; when CA is activated, it lowers the CBV and consequently the ICP. Furthermore, while REBOA is used in acute scenarios within the first hour after trauma, taking into account the formation of acute cerebral oedema after TBI is inappropriate [56, 57].

Arterial cerebral circulation may be involved in the response of ICP elevation to AO.

D’Ambra et al. investigated the phenomenon of transient ICP elevation after cross-clamping the descending aorta in normo-volemic rabbits with intact brain, and 30 s long reversible elevation of ICP. They attempted to explain the phenomenon by studying brain capacity to increase absorbing stroke volume after AO. However, the rise in ICP was not reproducible with phenylephrine-induced hypertension, nor associated with significant changes in CVP. In addition, reducing afterload with nitroprusside did not reduce the effect of AO in ICP elevation. They concluded that the transitory ICP elevation might be due to either an immediate, rapid reflex mechanism involving aortic arch receptors or neurohormonal responses related to spinal cord ischemia [16].

Strömholm et al. demonstrated, in one of their studies using MR in euvolemic pigs, that mean ventricular volume after 5 min aortic cross-clamping (ACC) of the descending aorta decreased to 8% ± 8 of the baseline value and to 91% ± 8 after 25 min. A further decrease in ventricular volume to 71% ± 10 of the baseline value was observed 5 min after declamping, before it returned to the baseline 25 min after declamping. He also observed that pMAP rose during ACC from a mean baseline value of 97 ± 17 mmHg to 152 ± 18mmHg before falling to 66 ± 6 mmHg after declamping without causing any oedema in cerebral grey or white matter, as recognised in the MR scans [21].

The dose of ICP delivered (ICP x time) may have a bearing on the development of brain injury [58, 59]. The dose of ICP delivered in the EICPG was increased by the activation of the REBOA. The elevated ICP clearly coincided with the occlusion of the aorta. This raised ICP dose may be harmful to the brain and adversely affect the outcome in the survivor. Åkerlund et al. have recently shown that an ICP of 18 ± 4 mmHg has a negative impact on outcome, and also that a higher ICP dose and compromised autoregulation cause a worse outcome [60].

### Cerebral perfusion pressure (CPP) / cerebral blood flow (CBF)

During the bleeding phase, we observed a decrease in CPP to low and potentially dangerous levels, especially in EICPG. CPP recovered after occlusion of the aorta in both groups. CPP levels after AO may generally be considered normal to slightly elevated in both groups. With CPP used as a surrogate measure for CBF, CBF did not seem to be compromised during the experiment after REBOA was applied, in either of the two groups.

In their study of tREBOA and pREBOA in the presence of an elevated ICP, the results reported by Edwards et al. are in all probability like ours, although the graphs and figures in their paper are somewhat hard to interpret. Our findings and those of Edwards et al. indicate that applying REBOA in severe HS restores CBF to normal values despite an existing HS, thus safeguarding the blood supply to the brain [43].

In terms of CPP, no difference was noted between the groups. The fall in CPP to very low levels before AO in EICPG can have grave metabolic consequences for the brain and cause irreparable injuries. Despite the rapid ICP elevation to pathological levels in the EICPG after the occlusion and the suspected alteration of cerebral autoregulation, CPP stayed stable within the normal range in both groups, with no difference between them.

Gelman et al. measured pMAP and CBF 20 min after total aorta cross-clamping of the descending aorta in an intact brain cortex of normo-volemic dogs. There was a rise in pMAP from 118 to 161 mmHg, but CBF was unchanged. This may reflect the preserved autoregulation of CBF as a response to the acute sudden elevation in pMAP [17].

#### Autoregulation

REBOA seems to be an effective tool to restore cerebral circulation in animlas with HS with normal and elevated ICP.

We see in our study recurrent positive mL-PRx values which indicate a disturbance in CA during the experiment in both groups during HS and AO. However, the disturbance is more pronounced in the EICPG.

The dramatic changes in CPP during the experiment need an effective autoregulation in order to mentain an adequate CBF.

Three different mechanisms are involved in CA, myogenic (BP), sympathic, and metabolic. These mechanisms work to adjust cerebrovascular resistence (CVR) by changing the diameter of cerebral vessels when CPP fluctuates. When CPP decreases the CVR decreses as well to prevent cerebral tissue hypoperfusion and or ischemia during hypotension. When CPP increases the CVR increses to protect cerebral circulation against hypervolumia, hyperemia during hypertension [61–63]. In addition, a static and a dynamic autoregulatory mechanism are involved continuasly to maintain an adequate CBF when BP fluctuates. Static autoregluation adjusts CVR to an adequate CPP value and determine how large changes in CPP can be compensated. Adynamic CA wich restores CBF after quick transient changes in CPP and determin how fast the the autoregularity compensation can be implemented [22, 64–69].

The early alteration of CA at the beginning of bleeding in the NICPG seems to recover sponeniusly even before AO. On the other hand the late deterioration of CA during bleeding phase in the EICPG seems to recover after AO.

A second worsening of the autoregulation started after 20 minuts of AO and lasted for 15 min in both grops before it recovers.

In the EICPG the second impairment of CA appears to coincide with the high pMAP which theoretically is outside of the autoregulatory window (50–150 mmHg), and can be explained by increase in CBF, as described by Hauerberg and Juhler in 1994 [70]. On the other hand the second CA impairment in NICPG is not associated with any dramatic changing in MAP or CPP and did recover sponteniosly.

A third impairment of CA was noticed in EICPG and lasted for 25 min before it recovered. During this period pMAP slowly decreases and was less than 150 mmHg.

The myogenic theory may explain the deterioration of CA in the first observed impairment episod in both groups (MAP < 50 mmHg) and in the second CA impairment in the EICPG (MAP > 150 mmHg), but not the second CA impairment in the NICPG or the third impairment in EICPG (< 50 MAP < 150 mmHg) where metabolic or sympathic hypothesis can be involved [61–63].

The associatiation of poor outcome with impaired CA was rapported in several studies [28, 71–75]. In 2021, Åkerlund et al. reported on the negative influence of compromised autoregulation on outcome in TBI. This could indicate that despite a restoration of CPP, the more impaired autoregulation in the EICPG may have an adverse impact on outcome in this group [60]. However, the correlation between PRx and outcome after brain injury is time dependent. PRx above 0,2 for more than 6 h is often associated with a fatal outcome [28, 76].

Applying REBOA for resuscitation of patients in HS with normal or increased ICP is lifesaving. Theoretically tREBOA alters CA, however this alteration is under short periods and reversible.

The definition of TBI, which is wide and non-specific, includes different brain injury types, such as intracerebral contusion and diffuse axonal injuries, presumably all with differing pathophysiology [77].

We chose a head-injury model, simulating an extra axial mass and creating an acute and controllable ICP elevation.

Our study has several limitations. First, the sample size is small, because of the strict regulation regarding ethical approval and the high cost of each experiment. Second, the study involves using resuscitation fluids and vasopressors that affect the blood circulation, haemodynamics and cardiac output, thus possibly altering cerebral haemodynamics. Third, the experimental set-up does not allow for late complications to be identified or the clinical outcome assessed. Fourth, the difference in pathophysiology in brain injuries owing to extra axial mass compared with brain structural lesions is not addressed. We were unable to determine the effects of the high ABP and ICP values on the bleeding from injured vessels. Fifth, we had a time resolution of 5 min for ICP and MAP. That’s why we chose to calculate the correlation using 6 time points of MAP and ICP for the calculation of one mLPRx value, then moving forward 5 min to repeat the calculation. Even though LPRx and mLPRx have the same principle for evaluation of autoregulation, using 6 consecutive points in mLPRx stills less accurate than using 20 consecutive points as in LPRx. Sixth, it would have had a great value if we included haematology to our experiment to illustrate eventual metabolic consequence during AO.

For a better understanding of the effect of tREBOA on the brain and its viability at cellular level, further studies are needed.

## Conclusion

Total REBOA restores the cerebral circulation, defined as CPP in animals in HS with normal or elevated ICP. Autoregulation is affected in both groups during HS and tREBOA but is more pronounced in animals with elevated ICP.

Restoration of cerebral circulation, achieved through tREBOA in animals in HS with normal or elevated ICP, is most probably lifesaving. The harm that an elevated CPP or dysfunctional autoregulation can potentially cause to the brain may be outweighed by the restoration of circulation to the brain achieved by activating the tREBOA in HS, irrespective of ICP level.

REBOA thus seems to be an effective tool for improving cerebral perfusion in HS that extends the crucial early window sometimes known as the “golden hour” for resuscitation even after a traumatic brain injury.

## Data Availability

No datasets were generated or analysed during the current study.

## Abbreviations

ABP: Arterial Blood Pressure
ACC: Aortic Cross-clamp
AO: Aortic Occlusion
CA: Cerebral Autoregulation
CBF: Cerebral Blood Flow
CH: Cerebral Hemodynamic
CPP: Cerebral Perfusion pressure
CVP: Central Venous Pressure
CVR: Cerebrovascular resistance
EICPG: Elevated Intracranial Pressure Group
HR: Heart rate
HS: Haemorrhagic shock
ICP: Intracranial Pressure
iREBOA: Intermittent Resuscitative Endovascular Balloon Occlusion of the Aorta
MAP: Mean Arterial Pressure
mLPRX: modified Long Pressure Reactivity Index
NICPG: Normal Intracranial Pressure Group
pMAP: Proximal Mean Arterial Pressure
pREBOA: Partial Resuscitative Endovascular Balloon Occlusion of the Aorta
PRX: Pressure Reactivity Index
REBOA: Resuscitative Endovascular Balloon Occlusion of the Aorta
T: Time
TBI: Traumatic Brain Injury
tREBOA: Total Resuscitative Endovascular Balloon Occlusion of the Aorta
